# Psychometric Properties of the French Version of the Physical Literacy in Children Questionnaire (PL-C Quest)

**DOI:** 10.3390/jfmk11040425

**Published:** 2026-10-16

**Authors:** Christophe Maïano, Alexandre J. S. Morin, Johanne April, Olivier Hue, Olivier Rey

**Affiliations:** 1 Department of Psychoeducation and Psychology, Université du Québec en Outaouais, Saint-Jérôme, QC J7Z 0B7, Canada; christophe.maiano@uqo.ca; 2 Cyberpsychology Laboratory, Department of Psychoeducation and Psychology, Université du Québec en Outaouais, Gatineau, QC J8X 3X7, Canada; 3 Substantive-Methodological Synergy Research Laboratory, Department of Psychology, Concordia University, Montreal, QC H4B 1R6, Canada; alexandre.morin@concordia.ca; 4 Optentia Research Unit, North-West University, P.O. Box 1174, Vanderbijlpark 1900, South Africa; 5 Department of Educational Sciences, Université du Québec en Outaouais, Saint-Jérôme, QC J7Z 0B7, Canada; johanne.april@uqo.ca; 6 Department of Physical Activity Sciences, Université du Québec à Trois-Rivières, Trois-Rivières, QC G9A 5H7, Canada; 7 Institute of Movement Sciences, Centre National de la Recherche Scientifique, Aix Marseille University, 13288 Marseille Cedex 09, France; olivier.rey@univ-amu.fr

**Keywords:** physical literacy, French, differential item functioning, age, body mass index, physical activity, sex

## Abstract

**Background and Objectives**: The main objective of this study was to examine the psychometric properties of a French adaptation of the Physical Literacy in Children Questionnaire (PL-C Quest). **Methods**: A sample of 196 French-speaking Canadian and French children (aged 5 to 12 years) participated in this study. **Results**: Matching the results from several previous studies, our results failed to support the adequacy of the *a priori* four-factor and hierarchical structure of the PL-C Quest. However, results from an exploratory factor analytic solution supported a 3-factor, 13-item solution of the PL-C Quest measuring (a) Physical Competencies; (b) Motivation and Enjoyment to be Physically Active; and (c) Self-Regulation, Risk, and Safety. Subsequent analyses revealed a lack of differential item functioning (DIF) as function of countries and children’s time spent in moderate to vigorous physical activities (MVPA) providing preliminary support for the comparability of scores across these groups. However, some limited evidence of DIF was found as function of children’s age (on one item), body mass-index (on two items) and sex (on one item). Furthermore, latent mean differences (favoring younger children and boys) were identified in (a) Self-Regulation, Risk, and Safety and (b) Physical Competencies as function of children’s age and sex, respectively. These findings should be interpreted cautiously and warrant replication in independent samples. Finally, results provided preliminary evidence for the convergent validity of the 13-item French version of the PL-C Quest in relation to parental ratings of children’s overall, cognitive, and motor physical literacy. **Conclusions**: Scores on the new 13-item French version of the PL-C Quest present acceptable psychometric properties in terms of composite reliability, factor validity, and minimal DIF, alongside preliminary support for convergent validity.

## 1. Introduction

Over the past 15 years, the concept of physical literacy has gained interest among researchers and practitioners [1,2,3]. First proposed by Whitehead [4], this concept has been found to be highly relevant in the fields of physical education, physical activity, leisure, and sport [2] and is most commonly defined as “*the motivation, confidence, physical competence, knowledge and understanding to value and take responsibility for engagement in physical activities for life*” [5] (para. 1). This concept is thus multidimensional and refers to the cognitive, physical, psychological, and social capabilities or predispositions required to be physically active over the lifespan [1,2].

The growing interest of researchers and practitioners in physical literacy has accelerated the development of various self-report questionnaires for school-aged children and adolescents [6,7]. The reviews of Barnett et al. [6] and Grauduszus et al. [7] identified six self-report questionnaires (i.e., Canadian Assessment of Physical Literacy; Chinese Assessment and Evaluation of Physical Literacy; Passport for Life; Physical Literacy Charting Tool; PLAYself; and Physical Literacy in Children Questionnaire) designed to assess the physical literacy of school-aged children (age 4–12 years) as a multidimensional construct. Moreover, Barnett et al. [6] concluded that the Physical Literacy in Children Questionnaire is one of the two instruments (the other one is the Passport for Life) whose scores seem to present the strongest reliability and validity.

### 1.1. The Physical Literacy in Children Questionnaire (PL-C Quest)

The PL-C Quest was developed based on the Australian Physical Literacy Framework (APLF), following an expert reference committee and a qualitative research method [8]. This pictorial self-report questionnaire includes 32 items, measuring cognitive (e.g., content knowledge and rules, 7 items), physical (e.g., object manipulation and muscular endurance, 13 items), psychological (e.g., motivation and self-regulation, 7 items), and social (e.g., relationship and collaboration, 5 items) dimensions of physical literacy. Importantly, two items from the physical dimension measuring moving with equipment, and two items from the social dimension measuring safety and risk, were formulated to be specific to countries with warm (one item for each dimension) and cold climates (one item for each dimension). A description of all elements measured by the PL-C Quest is presented in Table 1.

All items are rated on a four-point response scale. More precisely, for each item, two figures are first presented: one comprising a “bunny” character that is “*pretty good*” in a specific element (e.g., hopping), and another one, a second “bunny” character, that is “*not so good*” in this specific element. Then, the children are asked to choose the image that is most like them (i.e., the “bunny” character that is “*pretty good*” or “*not so good*”) and whether the image they selected is “*a lot*” or “*a bit*” like them.

Barnett, Mazzoli, Bowe et al. [9] examined the factor validity and reliability of scores obtained on the PL-C Quest among a sample of 669 Australian children (59% male) aged between 6.9 and 12.9 years. First, they examined the factor validity of PL-C Quest scores using a hierarchical confirmatory factor analysis (HO-CFA), including the four *a priori* factors (i.e., cognitive, physical, psychological, and social) and a second-order factor labeled physical literacy. This model resulted in an unacceptable level of fit to the data. To improve the fit of their model, the authors decided to include seven *ex post facto* correlated uniquenesses (CUs; 3 for the physical factor, 3 for the psychological factor, and 1 for the cognitive factor), arguing that these additions made sense theoretically. The inclusion of seven CUs helped to reach acceptable values for all fit indices (comparative fit index [CFI] = 0.910, Tucker–Lewis index [TLI] = 0.900, root mean square error of approximation [RMSEA] = 0.045). Although the resulting model was able to achieve a satisfactory level of fit to the data in the whole sample, the authors failed to find evidence supporting the adequacy of their model when considered separately in subsamples of boys and girls. Moreover, factor correlations between the four first-order factors were very high (0.73 to 1.00; *M* = 0.895), suggesting a lack of discriminant validity. However, the indices of scale score reliability reported by the authors were satisfactory (α = 0.72 to 0.94), and test–retest reliability calculated over an interval of two weeks to a month was moderate to high (intra-class correlations—ICC = 0.66 to 0.80).

### 1.2. Cross-Validation of the PL-C Quest in Other Languages or Cultures

To our knowledge, five studies tried to cross-validate the PL-C Quest, focusing either on the English version among British children [10] or on Chinese [11,12], Greek [13], and Spanish [14] adaptations.

The factor validity and reliability of scores on the English version of the PL-C Quest was assessed in a sample of 664 British children (8–11 years) [10]. Once again, the authors had to incorporate a large number of *ex post facto* CUs into their models (19 to 21, depending on the model). Additional results revealed latent correlations among the four first-order factors that were once again very high (0.40 to 0.99; *M* = 0.775), and a lack of weak invariance (i.e., indicating non-equivalent factor loadings) across subsamples of boys and girls, and as a function of school year (4 to 6). The authors finally report moderate estimates of scale score reliability (α = 0.70 to 0.90) and moderate-to-high estimates of test–retest reliability across a period of one week (ICC = 0.77 to 0.94).

Using a sample of 1870 children (4–12 years), Diao et al. [11] examined the psychometric properties of scores obtained on another Chinese adaptation of the PL-C Quest. Their results supported the adequacy of the *a priori* factor structure of scores obtained on this Chinese version of the instrument, as well as its strong (equivalent factor loadings and response thresholds) invariance as a function of sex, but not as a function of age. Once again, the authors report very high factor correlations (0.68 to 0.89; *M* = 0.813). The scale score reliability of scores obtained on this version was satisfactory (α = 0.81 to 0.86), and test–retest reliability was moderate to high across two weeks (0.71 to 0.86).

Using a sample of 642 children (6–12 years), Wu et al. [12] examined the psychometric properties of scores obtained on a Chinese adaptation of the PL-C Quest. Their results failed to support the adequacy of the *a priori* factor structure, which resulted in an unacceptable level of fit to the data and once again revealed very high correlations between the first-order factors (0.59 to 0.97; *M* = 0.828). Finally, the composite reliability of scores obtained on these factors was modest to good (ω = 0.65 to 0.89) and test–retest reliability was moderate to high across 12 days (ICC = 0.74 to 0.93).

Using a sample of 552 children (4–12 years), Kaioglou et al. [13] examined the psychometric properties of scores obtained on a Greek adaptation of the PL-C Quest. Their results failed to support the adequacy of the *a priori* factor structure, which resulted in an unacceptable level of fit to the data, with or without the inclusion of *ex post facto* CUs. Their results also failed to support the weak invariance of their model across subsamples of boys and girls. However, the authors report satisfactory estimates of scale score reliability (α = 0.72 to 0.83) and test–retest reliability over one week (ICC = 0.66 to 0.87). Finally, the authors provided evidence supporting the convergent validity of scores on their second-order factor in relation to another instrument measuring physical literacy.

Using a sample of 2004 children (8–14 years), Ortega-Benavent et al. [14] examined the psychometric properties of scores obtained on a Spanish adaptation of the PL-C Quest. Their results failed to support the adequacy of the *a priori* factor structure, which resulted in an unacceptable level of fit to the data. To obtain a solution associated with an adequate level of fit to the data, the authors had to incorporate 12 *ex post facto* CUs and to remove five items. Although the authors found evidence of weak invariance (equivalent factor loadings) across samples of boys and girls, this solution still resulted in very high factor correlations (0.47 to 1.00; *M* = 0.805). The scale score reliability of scores obtained on this final version was modest to acceptable (α = 0.57 to 0.73) and test–retest reliability was moderate to high across one week (ICC = 0.79 to 0.89). Finally, the authors provided evidence supporting the convergent validity of scores on this version of the instrument in relation to measures of perceived movement skill competence, motivation, social support, physical activity and perceived physical literacy.

### 1.3. The Present Study

Although informative, these studies present some relatively severe limitations. First, there is little consensus about the validity and generalizability of the four-factor (first-order or higher-order) structure of the PL-C Quest. Indeed, with a single exception limited to one Chinese version of this instrument [11], no study was able to find evidence supporting the adequacy of the *a priori* factor structure of this instrument, or to converge on a modified solution that was replicated across studies. Perhaps more importantly, the *ex post facto* incorporation of data-driven CUs should be avoided in research [15], and CUs should only be added in an *a priori* manner to account for clearly identifiable methodological artefacts (e.g., negative or parallel wording, unmodeled facets) [16,17]. This is not the case with the PL-C Quest, and the improper nature of these post hoc modifications is well-demonstrated by the fact that none of the previous studies was able to converge on a matching set of modifications.

Second, in most previous studies, the correlations between the four first-order factors of the PL-C Quest were very high (*M* = 0.775 to 0.895), sometimes reaching 1.00 [9,10,11,12,14]. These very high correlations call into question the relative independence of several of these factors. Importantly, inflated factor correlations may be explained by a lack of estimation of cross-loadings, which have been shown to help recover the true factor definition more accurately than confirmatory factor analysis (CFA) models excluding cross-loadings [16,18].

Third, very little evidence of measurement invariance (or lack of differential item functioning) has been provided in any of those studies, calling into question the possibility of using the PL-C Quest to conduct unbiased comparisons across subsamples of participants differing in terms of characteristics commonly examined in physical literacy research (e.g., age, sex, body mass index, and physical activity level).

Fourth, to our knowledge, only two studies have examined the convergent validity of scores obtained on the PL-C Quest [13,14], and both of those studies did so on the basis of a dubious factor analytic solution. Therefore, the convergent validity of scores obtained on this instrument remains essentially unknown, making it impossible to conclude whether this instrument truly captures physical literacy.

Finally, to our knowledge, no pictorial measure of physical literacy is currently available for French-speaking children. This lack of measure creates a significant obstacle to the implementation of cross-cultural studies on physical literacy involving French-speaking participants. Practically speaking, the availability of a French version will greatly facilitate comparison of physical literacy among French-speaking children living in European (e.g., Belgium, France, and Switzerland), American (Canada, Haiti, and French Guiana), and African (e.g., Algeria, Cameroon, Morocco, and Tunisia) countries.

The main objective of the present study was to address these limitations by examining the psychometric properties of a French adaptation of the PL-C Quest among French-speaking children recruited in Canada and in France. First, PL-C Quest was adapted to French. Second, the factor validity and reliability of scores on the *a priori* factor structure of this French adaptation were examined. However, should these *a priori* models fail to achieve adequate fit, an exploratory factor analytic solution (EFA) would then be estimated to see whether it may help resolve open questions related to the optimal factor structure of the PL-C Quest and whether cross-loadings can help achieve a clearer factor differentiation. Third, the presence of differential item functioning of PL-C Quest ratings as a function of age, country, sex, body mass index, and physical activity (moderate-to-vigorous intensity) was formally investigated. Fourth, the convergent validity of scores on the French adaptation of the PL-C Quest was examined in relation to measures of overall physical literacy as well as of cognitive and motor competence dimensions of physical literacy completed by the parents.

## 2. Materials and Methods

### 2.1. Participants

This study relies on a convenience sample of 196 French-speaking children (50% boys; aged 5 to 12 years; *M*_age_ = 8.59 years, *SD*_age_ = 1.90; BMI: 10.54 to 36.28 kg/m^2^, *M*_BMI_ = 16.48, *SD*_BMI_ = 3.20) recruited in elementary schools (95%) or via community organizations (5%) located in Canada (*N* = 123; 52% boys; *M*_age_ = 8.96 years; *M*_BMI_ = 17.77 kg/m^2^) and France (*N* = 73; 46.6% boys; *M*_age_ = 7.98 years; *M*_BMI_ = 15.96 kg/m^2^). Each week (Monday to Friday), these children spent, on average, 296.91 min (Canadian: *M* = 349.12; French: *M* = 272.59) engaged in physical activities of moderate intensity and 111.20 min (Canadian: *M* = 107.21; French: *M* = 113.05) engaged in physical activities of vigorous intensity. Children were not eligible to participate if they displayed characteristics likely to bias their responses to, or limit their ability to respond to, the PL-C Quest (i.e., developmental delay, neurological disorder, sensory or physical disabilities, and mobility assistance).

### 2.2. Measures

#### 2.2.1. Children Characteristics

Parents reported their child’s sex, age, height and weight. Children’s height and weight were used to estimate their BMI (Weight/Height^2^) expressed in kg/m^2^.

#### 2.2.2. Children’s Physical Literacy

Authorization to use and translate the PL-C Quest (version 1) [8] into French was obtained from the Australian Sports Commission in October 2021 (for Canada) and April 2022 (for France). The English version of the PL-C Quest was translated into French using standardized translation–back translation methods [19]. A first professional bilingual translator (not familiar with the PL-C Quest) translated the original English items into French. Then, the French-translated items were back-translated into English by a second independent professional bilingual translator who was also not aware of the original PL-C Quest items. Finally, the original (English) and back-translated items (French) were compared. Any discrepancies were resolved in a committee (i.e., comprising the two translators and four of the authors).

In the original PL-C Quest, two items are adapted differently for cold and warm countries (i.e., items moving with equipment and safety and risk). In the present study, the children were asked to complete the two versions of these items. The French translation of the items and the figures are copyrighted by the Australian Sports Commission and cannot be reproduced here. Authors seeking permission to use and to obtain a copy of the French version of the PL-C Quest should obtain authorization from the Australian Sports Commission. For more information regarding the PL-C Quest, see https://www.ausport.gov.au/physical_literacy/resources (accessed on 2 April 2026).

#### 2.2.3. Parental Assessment of Children’s Overall Physical Literacy

The French version of the physical literacy visual analog scale (VAS) section from the *PLAY*parent workbook [20] was used to obtain parental assessments of their child’s overall level of physical literacy. First, a definition of physical literacy was provided to parents, who were then invited to score *how their child’s physical literacy compares to someone with perfect physical literacy* [20] by placing a mark on a 100-millimetre VAS ranging from 0% (*not physically literate*; French: *aucune littératie physique*) to 100% (*perfect physical literacy;* French: *littératie physique parfaite*).

#### 2.2.4. Parental Assessment of the Cognitive and Motor Competence Dimensions of Children’s Physical Literacy

The cognitive and motor competence dimensions of children’s physical literacy were obtained from parents using 15 French items from the *PLAY*parent workbook [20]. The cognitive dimension comprises six items measuring parents’ assessment of their child’s confidence (e.g., *confidence to participate in physical activity*), motivation (e.g., *motivation to participate in physical activity and sport*), and comprehension (e.g., *knowledge related to healthy physical activity*) regarding physical activity and sport. The motor competence dimension comprises nine items measuring parents’ assessment of their child’s locomotor (e.g., ability to balance during movement) and object control (e.g., ability to use feet to kick or move objects) competencies. Parents rated each item using a 3-point scale ranging from 0 (low; French: *faible)* to 2 (high; French: *élevé*). In the present study, McDonald’s ω coefficients [21] for the cognitive and motor competence dimensions of physical literacy were 0.745 and 0.810, respectively.

#### 2.2.5. Time Spent Engaged in Moderate-to-Vigorous Physical Activities

Parents completed a French version of the proxy-report version of the Children’s Leisure Activities Study Survey (CLASS) [22]. Authorization to translate the CLASS into French was obtained in September 2021. The CLASS was adapted to French using the same procedure used for the PL-C Quest. The proxy-report version of the CLASS for parents includes a list of 30 physical activities. For each physical activity, parents were asked to indicate whether their child engages or not in this physical activity (yes or no) during a typical week (from Monday to Friday during the current school term). Parents reporting that their child was engaging in this physical activity were asked to report the frequency (number of times) and the total duration (in minutes or hours) during a typical week. As recommended by Telford et al. [22], ratings were converted into minutes per week (Monday to Friday) spent engaged in moderate-to-vigorous physical activities (MVPA).

### 2.3. Procedures

Authorization to conduct the study was obtained from the university research ethics committee of the first (#2022-2068) and last authors’ institution (#2023-06-15-008), as well as from school boards and principals. Participants were recruited from six elementary schools that agreed to participate or in the community via sport and leisure organizations and an advertisement in a local newspaper. No compensation was offered in France, whereas Canadian participants were eligible to win one of five gift certificates ($20 CAD).

Parents or legal representatives of all participating children actively provided their informed consent (paper or online) for their children’s participation. For parents of children recruited in schools, a consent form and information letter were sent by schools either online or in paper format. The signed paper consent form was returned to the school and recovered by a member of the research team, whereas the completed online consent form was directly received by the research team. For parents of children recruited in the community, the information letter and link to access the online consent form were posted on social media or sent by email by sport and leisure organizations. The completed online consent form was directly received by the research team. Consenting parents were then invited by email to access an online survey (hosted on LimeSurvey) about their children.

Once parental/legal consent was obtained, children were met individually (92.7% in Canada and 100% in France) at school or via Zoom (7.3% in Canada only) by a member of the research team. In this meeting, the objectives and procedures of the study were presented, and the child’s verbal assent to participate (or not) was obtained. To ensure consistency across data collection settings, a standardized administration protocol was implemented for in-person and Zoom assessments. All research team members underwent training (i.e., recruitment, testing, and response recording procedures), and consistency between study sites was further reinforced through a meeting involving representatives from each participating country.

After providing their verbal assent, children were invited to complete the PL-C Quest with the support of a trained member of the research team. During administration, interviewers read all instructions and questionnaire items aloud, while participants provided their responses independently. To preserve the objectivity of the assessment process, interviewers did not offer feedback, explanations, or guidance related to item content or response options. Their role was limited to repeating the standardized instructions when necessary and recording the responses provided by the children. The completion of the PL-C Quest generally took less than 10 min. Data was collected from April 2023 to May 2024.

### 2.4. Analyses

Given the ordinal nature and asymmetric thresholds of the response scales used in this study [23], analyses were performed using Mplus 8.11 [24] robust weighted least squares with mean and variance adjustment (WLSMV) estimator. The few missing responses at the item level (*M* = 2.63%) were handled with the missing data procedures implemented in Mplus in conjunction with the WLSMV estimator [25], allowing us to retain all participants.

The adequacy of the *a priori* four-factor (i.e., physical, psychological, cognitive, and social) and hierarchical (i.e., one second-order factor model and four first-order factors) solutions was verified using CFA. However, should all *a priori* models fail to achieve adequate fit, an EFA would then be estimated, using an oblique Geomin rotation procedure with an epsilon value of 0.5 to maximally reduce factor correlations [26,27]. A parallel analysis would then also be estimated using the nFactors (1000 randomly generated data sets) package v.2.4.1.2 [28] available in R v.4.4.2 [29] to determine the optimal number of factors.

The goodness-of-fit of all models was examined using (e.g., [30,31,32]): The CFI (≥0.90 and >0.95 reflect “*acceptable*” and “*excellent*” fit, respectively), the TLI (same thresholds as for the CFI), and the RMSEA (≤0.08 and ≤0.06 represent an “*acceptable*” and “*excellent*” fit, respectively). The composite reliability of PL-C Quest factors was estimated using the omega (*ω*) coefficient [21].

Formal tests of measurement invariance are hard to conduct when some of the groups that need to be compared are small (i.e., country and children’s sex in this study) and impossible to conduct as a function of naturally continuous variables for which arbitrary dichotomization results in a loss of valuable information (i.e., age, BMI, and MVPA in this study). In these situations, it is recommended to assess the impact of these characteristics on item responses and scores on the latent factors through tests of DIF implemented using a multiple indicators multiple causes (MIMIC) CFA model. In the present study, separate tests of DIF were performed for age, BMI, country, MVPA (time spent engaged in moderate physical activities and in vigorous physical activities were simultaneously included in the same MIMIC model), and sex. For each characteristic, these tests involved comparing the following models, in sequence [27,33]: (a) null effects model (paths from the age, BMI, country, MVPA, and sex to the latent factors and item responses were constrained to be zero); (b) saturated model (paths from age, BMI, country, MVPA, and sex to the item responses were freely estimated, while paths from age, BMI, country, MVPA, and sex to the latent factors were constrained to be zero); (c) factors-only model (paths from age, BMI, country, MVPA, and sex to the latent factors were freely estimated, while paths from age, BMI, country, MVPA, and sex to the item responses were constrained to be zero). Comparing the first two models (null and saturated) tests whether the predictor (age, BMI, country, MVPA, or sex) has an impact on the model, while comparing the last two models (saturated and factors-only) assesses whether this impact is limited to the factors or spreads to item responses as well. Model comparisons rely on the same guidelines typically used for tests of measurement invariance, such that ∆CFI and/or ∆TLI ≥ 0.01 and/or ∆RMSEA ≥ 0.015 are considered meaningful [34,35].

Finally, convergent validity was examined using a structural equation model (SEM) in which the PL-C Quest latent factors were correlated with observed scores (standardized) reflecting parental ratings of children’s overall, cognitive, and motor physical literacy. Importantly, tests of DIF and convergent validity were implemented using exploratory structural equation modeling (ESEM) [16].

## 3. Results

### 3.1. CFA and Hierarchical Order-CFA

The goodness-of-fit statistics of the *a priori* four-factor CFA and HO-CFA solutions are reported in the first section of Table 2. These results show that none of these models resulted in an acceptable level of fit to the data, leading us to reject these models. Power estimation was conducted based on the model’s data fit using an online calculator developed by Preacher and Coffman [36]. The Null RMSEA and alpha error were fixed to 0.08 and 0.05, and the following parameters were used: df, sample size, and value of the RMSEA. Based on the RMSEA associated with the CFA and HO-CFA solutions, power was 100% [36]. Based on the RMSEA associated with the 3-factor EFA solution, power was 100% [36].

### 3.2. Exploratory Factor Analysis

**Figure 1:**
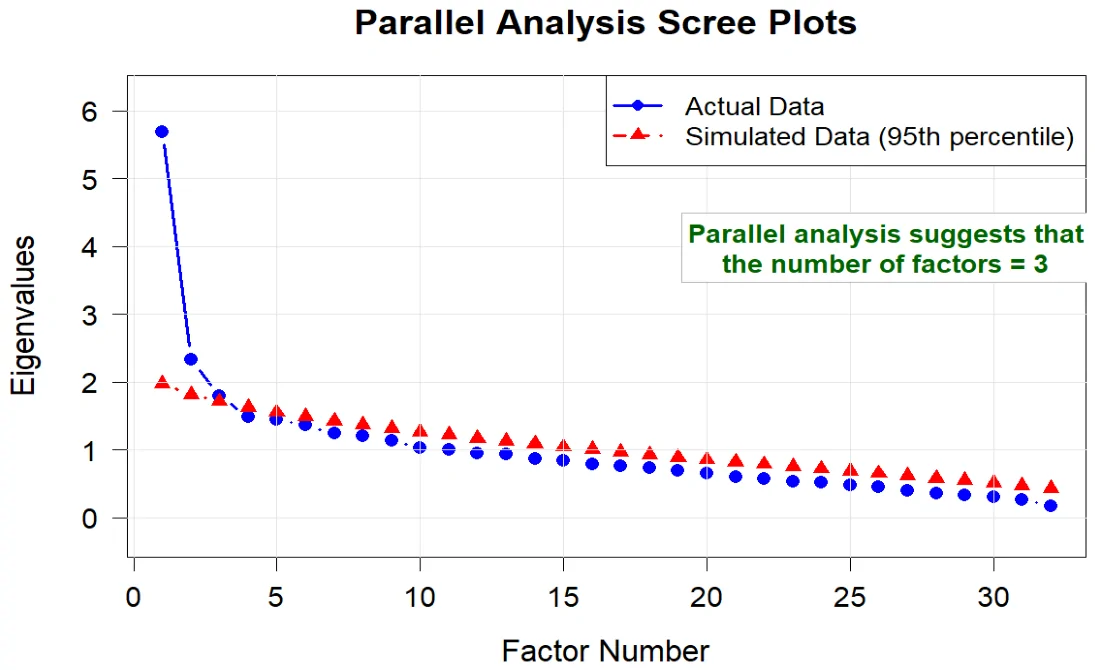
Parallel Analysis Scree Plot from the Exploratory Factor Analysis.

EFA solutions including one to eight factors were examined. Model fit indices, reported in the second section of Table 2, were unacceptable for the unidimensional model and acceptable to excellent for the remaining solutions including two to eight factors. The parallel analysis, reported in Figure 1, suggested that the data was best represented by a 3-factor solution. Parameter estimates from this 3-factor solution are reported in Table S1 of the online supplements and show that the main factor loading of 16 items (i.e., #2a, 2b, 6, 7, 8, 11, 16, 17, 19, 20, 21, 22, 24, 25, 27, and 28) was negligible and lower than 0.400. The first factor was primarily defined by seven items (i.e., #3, 4, 9, 10, 12, 15, and 29), of which five were from the *a priori* physical factor, one from the *a priori* psychological factor, and one from the *a priori* cognitive factor. This factor was labeled *Physical Competencies*. The second factor was primarily defined by five items (i.e., #1, 5, 13, 18, and 23), of which two were from the *a priori* physical factor, two from the *a priori* psychological factor, and one from the *a priori* cognitive factor. This factor was labeled *Motivation and Enjoyment to be Physically Active*. The third factor was primarily defined by four items (i.e., #14, 26, 30a, and 30b), of which one was from the *a priori* psychological factor and three from the *a priori* cognitive factor. This factor was labeled *Self-Regulation, Risk, and Safety*.

Although this solution had an excellent fit to the data, three items (i.e., #1 and #5 from the *Motivation and Enjoyment to be Physically Active* factor and #3 from the *Physical Competencies* factor) still displayed a negligible main loading lower than 0.400, leading us to re-estimate this model one last time from the final set of 13 items that retained satisfactory main loadings in all previous solutions. This model had an excellent fit to the data and all main loadings were higher than 0.400. The parameter estimates from the 13-item 3-factor solution are reported in Table 3. In this solution, all factors were defined by moderate-to-high factor loadings (Physical Competencies: λ = 0.484 to 0.674; *M*_λ_ = 0.590; Motivation and Enjoyment to be Physically Active: λ = 0.450 to 0.874; *M*_λ_ = 0.690; Self-Regulation, Risk, and Safety: λ = 0.502 to 0.850; *M*_λ_ = 0.662), with small cross-loadings (*M*_λ_ = 0.089). As shown in Table 3, latent correlations were all positive and modest (i.e., *M*_r_ = 0.136), but only statistically significant between Physical Competencies and Motivation and Enjoyment to be Physically Active. Finally, composite reliability coefficients were all satisfactory (ω *>* 0.700; *M*_ω_ = 0.767).

### 3.3. DIF Across Country, Age, BMI, MVPA, and Sex

The fit of the 13-item 3-factor models used to test the DIF of PL-C Quest responses as a function of country, age, BMI, MVPA, and sex are respectively reported in the third, fourth, fifth, sixth, and seventh sections of Table 2.

#### 3.3.1. Country

All three models had a comparable level of fit to the data, which is consistent with a complete lack of association between country of origin and item responses.

#### 3.3.2. Age

The saturated model had a better fit to the data than the null effects model, revealing a link between age and PL-C Quest responses. Moreover, the fit of the factors-only model was lower than that of the saturated model, suggesting DIF. Examination of the modification indices associated with the null effects and factors-only models, as well as of the freely estimated paths from the saturated model, suggested that this DIF was limited to item #12 and that the corresponding constraint needed to be relaxed (i.e., older children tended to score lower on this item than younger children). Once these effects were added to the factors-only model, the resulting model of partial DIF was supported by the data. Results from this model further showed that older children tended to present lower levels of Self-Regulation, Risk, and Safety (*β* = −0.409, SE = 0.072, *p* < 0.001) relative to younger children.

#### 3.3.3. BMI

The saturated model had a better fit to the data than the null effects model, revealing a link between BMI and PL-C Quest responses. Moreover, the fit of the factors-only model was lower than that of the saturated model, suggesting DIF. Examination of the modification indices associated with the null effects and factors-only models, as well as of the freely estimated paths from the saturated model, suggested that this DIF was limited to items #4 and #12 (i.e., children with a higher BMI tended to score lower on these items than their peers with a lower BMI). Once these effects were added to the factors-only model, the resulting model of partial DIF was supported by the data. Results from this model further showed a lack of relations between BMI and Physical Competencies (*β* = 0.042, SE = 0.093, *p* = 0.657), Motivation and Enjoyment to be Physically Active (*β* = −0.136, SE = 0.070, *p* = 0.054), and Self-Regulation, Risk, and Safety (*β* = −0.063, SE = 0.105, *p* = 0.550).

#### 3.3.4. MVPA

The saturated model had a better fit to the data than the null effects model, revealing a link between MVPA and PL-C Quest responses. Moreover, the fit of the factors-only model was comparable to that of the saturated model, supporting the idea that these associations are limited to the factors (no evidence of DIF). These results indicate that more time spent in vigorous physical activity tends to be associated with higher levels of Physical Competencies (*β* = 0.334, SE = 0.127, *p* = 0.009) and Motivation and Enjoyment to be Physically Active (*β* = 0.300, SE = 0.120, *p* = 0.012). However, no associations were found with time spent in moderate physical activity.

#### 3.3.5. Sex

The saturated model had a better fit to the data than the null effects model, revealing a link between sex and PL-C Quest responses. Moreover, the fit of the factors-only model was lower than that of the saturated model, suggesting DIF. Examination of the modification indices associated with the null effects and factors-only models, as well as of the freely estimated paths from the saturated model, suggested that this DIF was limited to item #23 (i.e., girls tended to score higher on this item than boys). Once these effects were added to the factors-only model, the resulting model of partial DIF was supported by the data. Results from this model further showed that girls tended to present lower levels of Physical Competencies (*β* = −0.336, SE = 0.077, *p* < 0.001) than boys.

### 3.4. Convergent Validity

The fit of the model used to assess convergent validity was excellent [χ^2^(72) = 75.042, CFI = 0.995, TLI = 0.992, RMSEA = 0.015 (90% CI = 0.000, 0.044)]. As reported in Table 4, the Physical Competencies and the Motivation and Enjoyment to be Physically Active factors of the PL-C Quest shared statistically significant, positive, and moderate relationships with parental ratings of children’s overall, cognitive, and motor physical literacy. In contrast, the Self-Regulation, Risk, and Safety factor of the PL-C Quest shared a statistically significant, negative and moderate relationship with parental ratings of children’s overall cognitive and motor physical literacy.

## 4. Discussion

This study sought to examine the psychometric properties of a French adaptation of the PL-C Quest among French-speaking children recruited in Canada and in France. Previous validation studies consistently failed to find evidence supporting the adequacy of the *a priori* four-factor (i.e., physical, psychological, cognitive, and social) and hierarchical (i.e., one second-order factor model and four first-order factors) structure of scores obtained on the PL-C Quest [9,10,12,13,14]. Consistent with these previous studies, our results also failed to support both *a priori* solutions, forcing us to rely on EFA to examine the structure of the PL-C Quest. These results suggested that a 3-factor, 13-item solution best represented the underlying structure of scores obtained on the PL-C Quest, capturing factors reflecting: (a) Physical Competencies; (b) Motivation and Enjoyment to be Physically Active; and (c) Self-Regulation, Risk, and Safety. Although this 13-item, 3-factor solution demonstrated satisfactory psychometric properties in the present sample, it remains important to acknowledge our results as providing tentative evidence for a preliminary representation of the PL-C Quest structure because it was both derived and evaluated within the same, relatively small, sample.

Although the final French PL-C Quest includes substantially fewer items than the original 32-item instrument, the retained items continued to reflect multiple aspects of physical literacy. Importantly, the reduction from four hypothesized domains to three empirically derived factors does not imply that the cognitive and social dimensions of physical literacy are absent from the present solution. Rather, several items originally associated with these domains were retained and loaded onto broader factors integrating content from multiple dimensions. Thus, the reduction in the number of items did not result in the complete removal of specific components of physical literacy, but rather in a more parsimonious organization of their content. Nevertheless, reducing the questionnaire from 32 to 13 items may have affected the breadth with which some aspects of physical literacy are represented. However, and more generally, our findings suggest that children may not conceptualize physical literacy according to the theoretical categories initially proposed in the PL-C Quest. Instead, their responses appear to reflect a more integrated and interconnected organization of physical literacy components. Importantly, the three retained factors remain conceptually aligned with core components of physical literacy, encompassing physical competence, motivation and engagement in physical activity, and self-regulatory behaviors related to participation in physical activity. Therefore, although the resulting factor structure differs from the four-domain model originally proposed for the PL-C Quest, it may be viewed as an alternative multidimensional organization of physical literacy attributes rather than merely a shortened data-driven questionnaire. Future research should continue to examine the extent to which this empirically derived factor structure corresponds to theoretical models of physical literacy across populations and cultures. In particular, the present findings raise the possibility that children may not differentiate the cognitive, social, psychological, and physical dimensions of physical literacy as distinctly as suggested by the APLF [8]. These dimensions may rather be experienced and reported as closely related overlapping aspects of a broader physical literacy construct.

Previous studies also provided very little evidence about the possibility of using the PL-C Quest to conduct unbiased comparisons across subsamples of participants. In addressing this limitation, our results first provided preliminary support for the use of the PL-C Quest to conduct comparisons across French and Canadian children, as no evidence of DIF was observed and no mean-level differences were found on any of the PL-C Quest factors across countries. Likewise, no evidence of measurement bias was found as a function of MVPA (i.e., no evidence of DIF). However, these findings should be interpreted with caution given the modest sample size, the presence of item-level DIF for other participant characteristics (i.e., age, BMI, and sex), and the absence of replication in an independent sample. In line with previous studies (e.g., [37,38]), our results also showed that children involved in higher levels of vigorous physical activity tended to demonstrate higher levels of Physical Competencies and Motivation and Enjoyment to be Physically Active.

In contrast, some evidence of DIF was found as a function of age, BMI, and sex. More precisely, our results show that children’s responses to items referring to: (a) speed physical ability (Item #12) were lower as they got older and had a higher BMI; (b) cardiovascular endurance ability (Item #4) were lower when they had a higher BMI; and (c) society and culture (Item #23) were higher for girls than for boys. Although these differences were limited to a small subset of items (confirming a lack of DIF associated with most items), these suggest that caution should still be used when conducting comparisons based on age, BMI and sex relying on observed (non-latent) scores. In these situations, it may be better to conduct these comparisons while excluding these items, or at least comparing the results obtained with and without them. In contrast, latent variable methods, such as those used in the present study, can be used to ensure unbiased comparisons while controlling for this partial DIF. In this regard, it is interesting to note that, in line with previous studies [9,11], our results showed that older children tended to present lower levels of self-regulation and of risk and safety, and that girls tended to present lower levels of Physical Competencies. However, no latent means differences were observed for BMI.

Finally, scores obtained on the 13-item French version of the PL-C Quest provided preliminary evidence of convergent validity through their associations with parental ratings of children’s overall, cognitive, and motor physical literacy. Because these findings were based exclusively on parental ratings, additional studies using objective indicators of physical literacy-related attributes and other validated physical literacy measures are needed to further strengthen evidence of convergent validity. More specifically, these moderate relations showed that parents’ and children’s perceptions are relatively well-aligned regarding cognitive and physical competence-related components of physical literacy. However, an unexpected finding was the negative association observed between the Self-Regulation, Risk, and Safety factor and parental ratings of children’s overall, cognitive, and motor physical literacy. One possible explanation is that parents may primarily base their judgments of their child’s physical literacy on more observable indicators, such as physical competence, participation in physical activity, and confidence in movement. In contrast, the Self-Regulation, Risk, and Safety factor may capture behavioral tendencies related to caution, risk management, and self-monitoring during physical activity that are less directly observable by parents. Consequently, higher parental perceptions of physical literacy may not necessarily correspond to higher levels of self-regulatory or safety-oriented behaviors, as reported by children.

Another possibility is that children who perceive themselves to be more physically competent, confident, and willing to engage in challenging activities may also be more inclined to take risks during play and physical activity, resulting in lower scores on items emphasizing safety-related behaviors. Therefore, these findings suggest that the Self-Regulation, Risk, and Safety factor may reflect aspects of physical literacy that are not fully captured by parental ratings. Given the exploratory nature of the present findings, future research will be needed to more thoroughly understand the role of Self-Regulation, Risk, and Safety within the broader construct of physical literacy and to further document the convergent validity of this factor using measures targeting similar behavioral attributes.

### Limitations and Directions for Future Research

Some limitations should be considered when interpreting these results. First, the extent to which the present findings can be generalized remains limited. Caution is particularly warranted when applying these results to the broader population of French-speaking children and youth, as well as to children from different linguistic and cultural backgrounds. This limitation stems from the fact that the psychometric evaluation of the French version of the PL-C Quest was based on a single sample of French and French-Canadian children. In addition, participants were recruited through convenience sampling. To strengthen evidence supporting the use of the PL-C Quest, future studies should focus on evaluating its psychometric properties in larger and more representative samples. Particular attention should be given to examining the measurement invariance of PL-C Quest scores across samples of other French-speaking children from different cultures/countries (e.g., Algeria, Belgium, Morocco, Switzerland, and Tunisia) and (b) children speaking other languages (e.g., English, Dutch, Italian, Malay, and Turkish).

Second, although the present findings provide initial support for a 3-factor solution of the PL-C Quest, the stability of this factor solution across samples remains to be established. Indeed, the present 3-factor solution was developed and tested using the same group of participants, and no independent cross-validation sample was available. As a result, future studies should examine the reproducibility of this factor solution in independent samples to strengthen confidence in its robustness and generalizability.

Third, children’s height and weight, as well as physical activity levels (CLASS), were obtained via parental reports rather than direct measurement. Therefore, BMI scores may have been underestimated compared with values derived from direct anthropometric assessments [39]. Similarly, parent-reported physical activity may be subject to reporting bias. Future studies should examine the extent to which our findings generalize to objective measures, such as direct anthropometric assessments and accelerometry-based measures of moderate-to-vigorous physical activity (MVPA).

Fourth, the test–retest reliability and longitudinal invariance of the French-version of the PL-C Quest should be more thoroughly examined in future longitudinal research. Lastly, the convergent validity of scores on the French-version of the PL-C Quest was essentially centered on dimensions of physical literacy (i.e., overall, cognitive, and motor competence), as reported by parents. Therefore, additional analyses are required to better document the convergent validity of the French-version of the PL-C Quest in relation to other physical literacy questionnaires as well as with objective measures of physical literacy (e.g., motor competence, physical fitness).

Fifth, although the WLSMV estimator and MIMIC modeling are generally considered appropriate for the analysis of ordinal indicators in samples of similar size, the overall sample remained relatively modest for the initial psychometric evaluation of a 32-item questionnaire. Furthermore, the relatively small country-specific subsamples warrant some caution in the interpretation of the DIF analyses. In addition, the final 13-item, 3-factor solution was both identified and evaluated within the same sample, and no independent cross-validation sample was available. Consequently, the stability and generalizability of this factor structure remain to be established. Replication of the present findings in larger and independent samples is therefore needed before the proposed 3-factor, 13-item solution can be considered firmly established.

Sixth, evidence supporting the convergent validity of scores obtained on the French version of the PL-C Quest was exclusively based on parental ratings of children’s physical literacy. Although parental reports provide a valuable external perspective, they remain subject to informant-specific biases and may not fully capture all components of children’s physical literacy. Consequently, the present findings should be interpreted as preliminary evidence of convergent validity. Future research should extend the present findings by examining associations between PL-C Quest scores and objective indicators of physical literacy, as well as with other validated measures of physical literacy.

Finally, a small proportion of Canadian participants (7.3%) completed the questionnaire via Zoom, whereas all French participants were assessed face-to-face. Although the proportion of remotely assessed participants was limited, future studies should examine whether administration mode influences responses to the PL-C Quest.

## 5. Conclusions

In conclusion, our results indicate that scores on the newly developed 13-item French version of the PL-C Quest present acceptable psychometric properties in terms of composite reliability, factor validity, and minimal DIF, alongside preliminary support for convergent validity. Additional results suggest that this shortened scale may be suitable for group-based comparisons across age, BMI, MVPA, and sex. Nevertheless, pending further research, adjustments (e.g., excluding the few items displaying DIF or contrasting comparisons with and without them) or methodological controls (i.e., latent variable models) should be implemented when conducting age-, BMI-, and sex-based comparisons. Finally, given the limitations of the present study, it is premature to recommend the use of the French version of the PL-C Quest in cross-linguistic/cultural and longitudinal studies. Future studies should seek to replicate the current findings in larger and more diverse independent samples.

## Figures and Tables

**Table 1:** Elements measured by the Physical Literacy in Children Questionnaire [8].

1. Movement skill	16. Self-perception
2a. Moving with equipment (cold climate countries)	17. Confidence
2b. Moving with equipment (warm climate countries)	18. Engagement and Enjoyment
3. Object manipulation	19. Connection to place
4. Cardiovascular endurance	20. Ethics
5. Muscular endurance	21. Relationships
6. Coordination	22. Collaboration
7. Stability/balance	23. Society and culture
8. Flexibility	24. Perceptual awareness
9. Agility	25. Content knowledge
10. Strength	26. Rules
11. Reaction time	27. Reasoning
12. Speed	28. Strategy and planning
13. Motivation	29. Tactics
14. Self-regulation (emotions)	30a. Safety and risk (cold climate countries)
15. Self-regulation (physical)	30b. Safety and risk (warm climate countries)

**Table 2:** Goodness-of-Fit Statistics for the Physical Literacy in Children Questionnaire.

Models	N^o^	Description	Wχ^2^	df	CFI	TLI	RMSEA	RMSEA 90% CI		CM	∆Wχ^2^	df	*p*	∆CFI	∆TLI	∆RMSEA
								LB	UB							
CFA	1-1	4 correlated factors	661.188 *	458	0.858	0.838	0.048	0.039	0.055	-	-	-	-	-	-	-
HO-CFA	1-2	1 s-order factor and 4 first-order factors	664.847 *	460	0.849	0.838	0.048	0.039	0.055							
EFA	2-1	1-factor—32 items	684.151 *	464	0.838	0.827	0.049	0.041	0.057	-	-	-	-	-	-	-
	2-2	2-factor—32 items	532.041 *	433	0.927	0.917	0.034	0.023	0.044	-	-	-	-	-	-	-
	2-3	3-factor—32 items	461.477 *	403	0.957	0.947	0.027	0.011	0.038	-	-	-	-	-	-	-
	2-4	4-factor—32 items	419.801 *	374	0.966	0.955	0.025	0.000	0.037	-	-	-	-	-	-	-
	2-5	5-factor—32 items	381.511	346	0.974	0.963	0.023	0.000	0.036	-	-	-	-	-	-	-
	2-6	6-factor—32 items	344.566	319	0.981	0.971	0.020	0.000	0.035	-	-	-	-	-	-	-
	2-7	7-factor—32 items	311.634	293	0.986	0.977	0.018	0.000	0.034	-	-	-	-	-	-	-
	2-8	8-factor—32 items	282.919	268	0.989	0.980	0.017	0.000	0.034	-	-	-	-	-	-	-
	2-9	3-factor—16 items	84.879	75	0.987	0.979	0.026	0.000	0.050							
	2-10	3-factor—13 items	36.473	42	1.000	1.000	0.000	0.000	0.038	-	-	-	-	-	-	-
DIF: Country	3-1	Null effects	55.315	55	0.999	0.999	0.005	0.000	0.045	-	-	-	-	-	-	-
	3-2	Saturated	36.595	42	1.000	1.000	0.000	0.000	0.038	3-1	15.854	13	0.257	+0.001	+0.001	−0.005
	3-3	Factors-only	46.184	52	1.000	1.000	0.000	0.000	0.036	3-1	4.995	3	0.172	+0.001	+0.001	−0.005
DIF: Age	4-1	Null effects	94.138 *	55	0.938	0.897	0.060	0.039	0.081	-	-	-	−	−	−	−
	4-2	Saturated	36.019	42	1.000	1.000	0.000	0.000	0.037	4-1	42.112	13	<0.001	+0.062	+0.103	−0.060
	4-3	Factors-only	59.457	52	0.988	0.979	0.027	0.000	0.055	4-1	15.218	3	0.001	+0.050	+0.021	−0.033
	4-4	Partial DIF	47.928	51	1.000	1.000	0.000	0.000	0.041	4-2	11.010	9	0.275	0.000	0.000	0.000
DIF: BMI	5-1	Null effects	65.634	55	0.983	0.972	0.031	0.000	0.057	-	-	-	−	−	−	−
	5-2	Saturated	34.874	42	1.000	1.000	0.000	0.000	0.034	5-1	23.192	13	0.040	+0.017	+0.028	−0.031
	5-3	Factors-only	58.392	52	0.990	0.982	0.025	0.000	0.054	5-1	5.412	3	0.144	+0.007	+0.010	−0.006
	5-4	Partial DIF	45.994	50	1.000	1.000	0.000	0.000	0.040	5-2	9.679	8	0.288	0.000	0.000	0.000
DIF: MVPA	6-1	Null effects	83.359	68	0.975	0.961	0.034	0.000	0.056	-	-	-	−	−	−	−
	6-2	Saturated	39.539	42	1.000	1.000	0.000	0.000	0.044	6-1	38.058	26	0.060	+0.025	+0.039	−0.034
	6-3	Factors-only	53.773	62	1.000	1.000	0.000	0.000	0.031	6-1	15.740	6	0.015	+0.025	+0.039	−0.034
DIF: Sex	7-1	Null effects	102.538 *	55	0.926	0.877	0.066	0.046	0.086	-	-	-	−	−	−	−
	7-2	Saturated	38.073	42	1.000	1.000	0.000	0.000	0.041	7-1	47.227	13	<0.001	+0.074	+0.123	−0.066
	7-3	Factors-only	64.634	52	0.980	0.965	0.035	0.000	0.060	7-1	16.994	3	<0.001	+0.054	+0.088	−0.031
	7-4	Partial DIF	46.869	51	1.000	1.000	0.000	0.000	0.039	7-2	8.494	9	0.485	0.000	0.000	0.000

*Note*. Wχ^2^ = robust weighted least square (WLSMV) chi-square; df = degrees of freedom; CFI = comparative fit index; TLI = Tucker–Lewis index; RMSEA = root mean square error of approximation; 90% CI = 90% confidence interval of the RMSEA; CM = comparison model; LB = lower bound; UB = upper bound; ∆Wχ^2^ = WLSMV chi square difference test (calculated with the Mplus DIFFTEST function); ∆ = change from the previous model; CFA = confirmatory factor analyses; HO-CFA = hierarchical order CFA; EFA = exploratory factor analysis; DIF = differential item functioning; BMI = body mass index; MVPA = moderate-to-vigorous physical activities. * *p* ≤ 0.05.

**Table 3:** Standardized Parameter Estimates from the Modified 3-Factor Exploratory Structural Equation Model of the Physical Literacy in Children Questionnaire (13 items).

Items	Physical Competencies (λ)	Motivation and Enjoyment to be Physically Active (λ)	Self-Regulation, Risk, and Safety (λ)	δ
4	**0.674**	0.144	*−0.084*	0.472
9	**0.544**	*0.105*	*−0.031*	0.662
10	**0.607**	*−0.120*	*0.019*	0.657
12	**0.647**	*0.127*	*−0.094*	0.518
13	*−0.014*	**0.874**	*0.008*	0.241
14	*0.116*	*0.109*	**0.502**	0.702
15	**0.583**	0.158	*0.133*	0.553
18	*0.027*	**0.746**	*−0.049*	0.433
23	*0.061*	**0.450**	*0.070*	0.769
26	*0.011*	*−0.087*	**0.713**	0.491
29	**0.484**	*0.142*	0.209	0.647
30a	*−0.127*	*−0.022*	**0.850**	0.275
30b	*0.128*	*0.128*	**0.581**	0.602
ω	0.781	0.748	0.772	
Motivation and Enjoyment to be Physically Active	0.286	−		
Self-Regulation, Risk, and Safety	*0.062*	*0.060*	−	

Note. λ = factor loadings; δ = uniquenesses; ω = McDonald’s omega. Main loadings are in bold. Non-significant parameters are underlined and italicized.

**Table 4:** Convergent Validity from the 3-Factor Exploratory Factor Analytic Representation of the Physical Literacy in Children Questionnaire.

	**Physical Competencies**	**Motivation and Enjoyment to be Physically Active**	**Self-Regulation, Risk, and Safety**
Parents’ assessment of children’s			
Overall level of physical literacy	0.405 **	0.280 **	−0.224 *
Cognitive dimension of physical literacy	0.320 **	0.413 **	−0.269 **
Motor competence dimension of physical literacy	0.315 **	0.357 **	−0.338 **

*Note*. PL-C Quest = Physical Literacy in Children Questionnaire. * *p* ≤ 0.05; ** *p* ≤ 0.01.

## Data Availability

The data is unavailable due to ethical restrictions.

## Supplementary Materials

The following supporting information can be downloaded at https://www.mdpi.com/article/10.3390/jfmk11040425/s1. Table S1: Three-Factor Exploratory Factor Analysis Solution for the Initial 32-item Version of the PL-C Quest.

## Abbreviations

The following abbreviations are used in this manuscript:

PL-C Quest	Physical Literacy in Children Questionnaire
HO-CFA	Hierarchical confirmatory factor analysis
CFA	Confirmatory factor analysis
EFA	Exploratory factor analysis
BMI	Body mass index
VAS	Visual analog scale
CLASS	Children’s Leisure Activities Study Survey
MVPA	Moderate-to-vigorous physical activities
WLSMV	Robust weighted least squares with mean and variance adjustment
CFI	Comparative fit index
TLI	Tucker–Lewis index
RMSEA	Root mean square error of approximation
DIF	Differential item functioning
MIMIC	Multiple indicators multiple causes
ESEM	Exploratory structural equation modeling
RMSEA 90% CI	90% confidence interval of the RMSEA
CM	Comparison model
