# Supplementary material for: Psychometric Properties of the French Version of the Physical Literacy in Children Questionnaire (PL-C Quest)
Source: J Funct Morphol Kinesiol. 2026 Oct 16;11(4):425. doi: 10.3390/jfmk11040425 (PMC13692324; doi:10.3390/jfmk11040425)
Supplement: Supplementary file 1 [file jfmk-11-00425-s001.zip › jfmk-4559394-supplementary.pdf]

Supplementary Materials for:

Psychometric Properties of the French Version of the Physical Literacy in Children Questionnaire (PL-C Quest)

Table S1. *3-Factor Exploratory Factor Analysis Solution for the Initial 32-item Version of the PL-C Quest*

Items	Factor 1 (λ)	Factor 2 (λ)	Factor 3 (λ)	δ
1	*.114*	**.445**	*-.028*	.752
2a	*.158*	.288	*-.167*	.853
2b	.255	.250	*.066*	.805
3	**.404**	*.096*	*.001*	.795
4	**.635**	*.138*	-.148	.523
5	.170	**.424**	*-.011*	.735
6	*-.029*	.302	*.082*	.901
7	.227	*.196*	*.029*	.869
8	*-.075*	.240	.263	.868
9	**.591**	*.052*	*-.001*	.623
10	**.612**	*-.119*	*.009*	.668
11	.324	.260	*.072*	.740
12	**.629**	.173	*-.143*	.505
13	*-.054*	**.805**	*.008*	.382
14	*.101*	*.113*	**.483**	.701
15	**.555**	.158	*.144*	.541
16	.308	.306	*.097*	.706
17	.229	.340	*-.143*	.776
18	*.002*	**.745**	*-.029*	.451
19	.248	*.110*	.327	.758
20	.236	*.170*	.199	.817
21	*.100*	*.012*	.381	.829
22	.299	*.054*	.241	.809
23	*-.059*	**.549**	*.105*	.694
24	.294	*.051*	.316	.763
25	.323	.326	.230	.602
26	*-.019*	-.183	**.746**	.457
27	*.134*	.392	.289	.652
28	.246	.222	.189	.781
29	**.531**	*.016*	.251	.603
30a	-.244	*.037*	**.860**	.265
30b	*.114*	*.032*	**.546**	.658

Note. PL-C Quest = Physical Literacy in Children Questionnaire; λ = factor loading; δ = uniquenesses. Non-significant parameters are underlined and italicized.
